# The variability of MR axon radii estimates in the human white matter

**DOI:** 10.1002/hbm.25359

**Published:** 2021-02-12

**Authors:** Jelle Veraart, Erika P. Raven, Luke J. Edwards, Nikolaus Weiskopf, Derek K. Jones

**Affiliations:** ^1^ Bernard and Irene Schwartz Center for Biomedical Imaging, Department of Radiology New York University Grossman School of Medicine New York New York USA; ^2^ CUBRIC, School of Psychology Cardiff University Cardiff UK; ^3^ Department of Neurophysics Max Planck Institute for Human Cognitive and Brain Sciences Leipzig Germany; ^4^ Felix Bloch Institute for Solid State Physics, Faculty of Physics and Earth Sciences Leipzig University Leipzig Germany; ^5^ Mary MacKillop Institute for Health Research Australian Catholic University Melbourne Victoria Australia

**Keywords:** axon diameter, diffusion MRI, reproducibility, variability

## Abstract

The noninvasive quantification of axonal morphology is an exciting avenue for gaining understanding of the function and structure of the central nervous system. Accurate non‐invasive mapping of micron‐sized axon radii using commonly applied neuroimaging techniques, that is, diffusion‐weighted MRI, has been bolstered by recent hardware developments, specifically MR gradient design. Here the whole brain characterization of the effective MR axon radius is presented and the inter‐ and intra‐scanner test–retest repeatability and reproducibility are evaluated to promote the further development of the effective MR axon radius as a neuroimaging biomarker. A coefficient‐of‐variability of approximately 10% in the voxelwise estimation of the effective MR radius is observed in the test–retest analysis, but it is shown that the performance can be improved fourfold using a customized along‐tract analysis.

## Graphical abstract

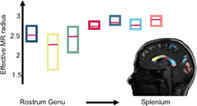

We demonstrate a good to excellent reliability in the quantification of micron‐sized effective MR radii using *in vivo* human dMRI. As a result, we were able to observe the subtle inter‐ and along‐tract variability in major white matter bundles.

## 1 INTRODUCTION

The white matter of the central nervous system is an intricately organized system of neural pathways that link together anatomical areas to create functional circuits. These neural pathways are formed by bundles of densely packed micrometer‐thin axons that are responsible for the transfer of information. The caliber of the axon and the presence of myelin are the most important morphological determinants that control the conduction velocity of action potentials (Drakesmith et al., 2019; Waxman, 1980).

Axons are susceptible to a variety of insults, in part due to their unique morphology and energy requirements (Conforti, Gilley, & Coleman, 2014; Perge, Koch, Miller, Sterling, & Balasubramanian, 2009; Wu, Williams, & Nathans, 2014). Axonal degeneration and/or dysfunction has been linked to physical trauma, oxygen and glucose deprivation, inflammation, and gene mutations (reviewed by Stassart, Möbius, Nave, & Edgar, 2018). Axonal degeneration is an early feature of neurodegenerative diseases, such as Alzheimer's disease (e.g., Blazquez‐Llorca et al., 2017) and multiple sclerosis (e.g., Evangelou et al., 2001). In injury, axonal loss may occur depending on the extent of injury in affected white matter tracts (reviewed in Nashmi & Fehlings, 2001). There is also evidence to suggest that altered features of axons, such as distributions of axon calibers or focal swellings, may contribute to various pathologies (Bartzokis, 2011) and neurodevelopmental disorders (Raven et al., 2020; Stassart et al., 2018). For example, in an animal model of Angelman syndrome, a rare genetic disorder linked to autism, widespread reductions in white matter volumes were linked to reduced numbers of axons with large radii (Judson et al., 2017). Similar observations were made in children with autism spectrum disorder (ASD), where electron microscopy identified a lower percentage of large‐radii axons in the corpus callosum compared to age‐matched typical developing children (Wegiel et al., 2018). Zikopoulos and Barbas (2010) reported a significantly lower relative density of extra‐large axons in prefrontal white matter in brains of adults with ASD. These *postmortem* studies demonstrate the potential of the noninvasive quantification of axon radii (including the ability to perform longitudinal assessment in the same individual), for understanding neuropathology in clinical research and, potentially, diagnostics. Critically, and as discussed below, diffusion‐weighted MRI methods for mapping axon diameter are more sensitive to larger axons than smaller axons, and so this preferential loss of axons with large radii in various disorders means that the technique holds great promise as a biomarker.

Diffusion‐weighted MRI (dMRI) is a particularly relevant neuroimaging modality to probe cellular features, far below the resolution of the imaging experiment (Alexander et al., 2010; Assaf Blumenfeld‐Katzir, Yovel, & Basser, 2008; Fan et al., 2020; Huang et al., 2020; McNab et al., 2013; Romascano et al., 2020; Sepehrband, Alexander, Kurniawan, Reutens, & Yang, 2016; Veraart et al., 2020). Indeed, dMRI is sensitive to a wide range of tissue microstructural parameters because the signal is sensitized to the micrometer length scale of the diffusion of water molecules (Tanner, 1979). The development of axon diameter mapping using dMRI has been challenged by various confounding factors that resulted in a significant and contested overestimation of the axon radius using MRI (Burcaw, Fieremans, & Novikov, 2015; Horowitz et al., 2015; Innocenti, Caminiti, & Aboitiz, 2015; Lee et al., 2018; Lee, Jespersen, Fieremans, & Novikov, 2020; Nilsson, Lasič, Drobnjak, Topgaard, & Westin, 2017). Novel insights in biophysical modeling and hardware developments improved the accuracy of axon radius mapping significantly (Jones et al., 2018; McNab et al., 2013; Veraart et al., 2020). However, MR axon radius mapping cannot replace *in vivo* microscopy. First, in the absence of strong *a priori* distributional assumptions (Assaf et al., 2008; Sepehrband et al., 2016), the information obtained from dMRI is typically limited to a single scalar representing the entire underlying axon distribution (e.g., Alexander et al., 2010; Fan et al., 2020; Veraart et al., 2020). Second, this scalar is strongly biased towards the largest axons, with nearly no sensitivity to the bulk of the axons (Burcaw et al., 2015; Nilsson et al., 2017). Note that we here adopt the term “effective MR radius” to refer to the MR‐derived axon radius (Veraart et al., 2020). In contrast, microscopy is a highly reproducible technique for the extraction of the bulk of the smaller axons, but suffers from a poor precision in the quantification of the underrepresented larger axons (Aboitiz, Scheibel, Fisher, & Zaidel, 1992).

Large‐radii axons are nevertheless important in brain function, especially in mammals with increased brain size. First, axons of large radii are capable of more rapid conduction, which is advantageous for time‐sensitive processes. Second, it has been hypothesized that the large‐radius axons of long‐range neurons are essential to maintaining neural synchrony (Buzsáki, Logothetis, & Singer, 2013). However, large‐radii axons come at a disproportionate cost in terms of energy use and spatial constraints (Knowles, 2017; Perge et al., 2009). Histological studies have extensively reported axon radii to be in the range 0.25–1 μm for human brain (Aboitiz et al., 1992; Caminiti, Ghaziri, Galuske, Hof, & Innocenti, 2009; Liewald, Miller, Logothetis, Wagner, & Schüz, 2014; Tang, Nyengaard, Pakkenberg, & Gundersen, 1997), with only 1% of all axons having a radius greater than 1.5 μm (Caminiti et al., 2009). Indeed, despite different white matter tracts having very different lengths and interconnecting entirely different functional circuits, in common they share a skewed axon radius distribution, characterized by mostly thin axons (Aboitiz et al., 1992; Caminiti et al., 2009; Liewald et al., 2014; Perge et al., 2009; Tomasi, Caminiti, & Innocenti, 2012). The radii of the bulk of smaller axons do not vary significantly across mammals with varying brain size. However, it has been shown that larger brains have more large axons and an increased maximal radius (Olivares, Montiel, & Aboitiz, 2001; Schüz & Preiβl, 1996). Notably, this observation promotes MR axon diameter mapping in the human brain.

In recent work, the accuracy of effective MR radius estimation using dMRI was assessed through a comparison of microscopy and MRI in fixed white matter tissue of the rodent brain (Veraart et al., 2020). In addition, the feasibility of the technique for *in vivo* human MRI has already been established by comparing MR‐derived values in the human corpus callosum to values reported in the literature (Fan et al., 2020; Veraart et al., 2020). Here, we will: (a) present the whole brain characterization of the effective MR radius; and (b) evaluate the inter‐ and intra‐scanner test–retest reliability (repeatability and reproducibility) to promote the further development of the effective MR radius as a neuroimaging biomarker.

## 2 THEORY AND METHODS

### 2.1 Axon diameter mapping

In an experimental regime in which the extra‐cellular signal can be assumed to be fully suppressed, the spherically‐averaged signal ${\overset{\circ }{S}}_{\mu }$ can be modeled as:

(1) $${\overset{\circ }{S}}_{\mu }=\beta \frac{S_{c}^{⊥}\left(r|q,\delta ,\Delta \right)}{\sqrt{b}},$$

with *b* = *q*^2^*δ*^2^(Δ − *δ*/3) (Veraart et al., 2020). The *b*‐value quantifies the diffusion‐weighting strength for the monopolar Stejskal‐Tanner pulse sequence with diffusion gradient duration *δ* and diffusion gradient separation Δ (Le Bihan et al., 1986; Stejskal, 1965). The prefactor $\beta \approx \frac{f}{\sqrt{D_{a}^{∥}}}$ is a function of the intra‐axonal signal fraction *f* and the parallel intra‐axonal diffusivity $D_{a}^{∥}$. The radial signal attenuation is modeled using the Gaussian phase approximation (Murday & Cotts, 1968) of the signal from protons trapped inside a cylinder with radius *r*:

(2) $$\begin{array}{l} \ln S_{c}^{⊥}\left(r\right)=-\frac{2q^{2}r^{4}}{D_{0}}\sum_{m=1}^{\infty }\frac{t_{c}}{\alpha_{m}^{6}\left(\alpha_{m}^{2}-1\right)}\cdot \\ \;\left[2\alpha_{m}^{2}\frac{\delta }{t_{c}}-2+2e^{-\alpha_{m}^{2}\delta /t_{c}}+2e^{-\alpha_{m}^{2}\Delta /t_{c}}-e^{-\alpha_{m}^{2}\left(\Delta -\delta \right)/t_{c}}\right)\\ \;\left(-e^{-\alpha_{m}^{2}\left(\Delta +\delta \right)/t_{c}}\right]+O\left(q^{4}\right), \end{array}$$

where *q* = *γG* is the diffusion‐weighting wave vector with *γ* the gyromagnetic ratio for protons and *G* the gradient strength (Neuman, 1974; van Gelderen, DesPres, van Zijl, & Moonen, 1994). Furthermore, *D*_0_ is the diffusivity of the axoplasm, *α*_*m*_ is the *m*^th^ root of d*J*_1_(*α*)/d*α* = 0, and *J*_1_(*α*) is the Bessel function of the first kind. Here, *t*_*c*_ = *r*^2^/*D*_0_ is the diffusion time across the cylinder. The applicability of the Gaussian phase approximation in the context of axon diameter mapping has been studied in detail by Fan et al. (2020).

Under the assumption that the extra‐cellar water is relatively mobile, that is, the extra‐cellular diffusivity is nonzero in the radial direction, then its spherically‐averaged signal decays exponentially fast, much faster than the intra‐axonal signal that decays as $1/\sqrt{b}$. In Veraart, Fieremans, and Novikov (2019), it has been observed that from *b* = 6  ms/μm^2^ upwards the extra‐cellular signal does not contribute significantly to the dMRI signal decay in the healthy human white matter. In comparison, earlier simulation studies reported the cut‐off for extra‐cellular signal to be as low as *b* = 3  ms/μm^2^ (Raffelt et al., 2012). In this work, we adopt the higher threshold to minimize the impact of this potential confound.

The associated software is available for download (Veraart & Novikov, 2019).

### 2.2 Effective MR radius

In the wide pulse limit (Neuman, 1974), *r* in Equations (1) and (2) denotes the effective MR radius, a scalar metric that represents the entire axon radius distribution captured within a single voxel, with minimal loss of accuracy (Veraart et al., 2020). As explained in detail in Burcaw et al. (2015) and Veraart et al. (2020), *r*^4^ equals the ratio between the sixth‐order and second order moment of the axon radii distribution. The sixth‐order in the numerator arises from the combination of biquadratic relation between $\ln\;S_{c}^{⊥}\left(r\right)$ and *r* (Neuman, 1974), and of the subsequent volume‐weighting that emphasizes the thickest axons by an extra quadratic factor (Alexander et al., 2010; Packer & Rees, 1972). Therefore, the effective MR radius is heavily weighted by the largest axons within the voxel or, more specifically, the largest Martin's radius in case of non‐cylindrical axons (Andersson et al., 2020).

### 2.3 Diffusion MRI experiments

Five healthy adult volunteers were recruited and data were collected on two different scanning sessions for each participant with exactly the same imaging protocol on a Siemens Connectom 3T MR scanner using a 32‐channel receiver coil and 300 mT/m gradient coils at the Cardiff University Brain Research Imaging Centre (CUBRIC), UK. For each volunteer, the two scanning sessions (“test” and “retest”) were performed one after the other, with a short break (10 min) between them. For both sessions, the subjects were positioned by the same operator. For one subject, the experiment was repeated 8 weeks after the initial experiments on an identical Siemens Connectom 3T MR scanner at the Max Planck Institute for Human Cognitive and Brain Science (MPI‐CBS), in Germany using identical imaging protocols.

Data were collected under the approval of: (a) the Cardiff University School of Psychology Ethics Committee (CUBRIC); and (b) the ethics committee of the Medical Faculty at Leipzig University. The participants gave written informed consent before participation in the study.

Diffusion weighting was applied with *b* = 0.5,1,2.5,6, and 30 ms/μm^2^, for 30, 30, 30, 120, and 240 gradient directions that were isotropically distributed on a sphere, respectively (Jones, Horsfield, & Simmons, 1999). The diffusion gradients were characterized by Δ/*δ* = 30/15 ms and maximal gradient amplitude of 273  mT/m—see Supplementary material for considerations regarding this protocol design. The following scan parameters were kept constant: TR/*T*_E_ : 3500/66 ms, matrix: 88 × 88, and 54 slices with a spatial resolution of 2.5 × 2.5 × 2.5  mm^3^. Data were acquired with a multi‐band blipped‐CAIPI accelerated (SMS = 2) EPI sequence with additional GRAPPA acceleration (*R* = 2) (Setsompop et al., 2012). Partial Fourier encoding was turned off. In addition, non‐diffusion‐weighted images were acquired with the same (*N* = 23) and reversed phase encoding (*N* = 10) for susceptibility‐induced geometrical distortion correction.

The axon diameter mapping pipeline employed here only uses the diffusion‐weighted data with *b* = 6 and 30 ms/μm^2^, which were acquired in 24 min per session. The additional data with *b* ≤ 2.5 ms/μm^2^ were only used for diffusion tensor imaging (DTI; Basser, Mattiello, & LeBihan, 1994) and diffusion kurtosis imaging (DKI; Jensen, Helpern, Ramani, Lu, & Kaczynski, 2005) analysis. The total scan time, covering both sessions, was 55 min.

### 2.4 Image processing

The diffusion‐weighted images were corrected for Gibbs ringing (Kellner, Dhital, Kiselev, & Reisert, 2016), geometric susceptibility‐ and eddy current distortions and subject motion (Andersson & Sotiropoulos, 2016), signal outliers (Andersson, Graham, Zsoldos, & Sotiropoulos, 2016). The *b*‐values were scaled to account for the gradient nonlinearities (Bammer et al., 2003; Rudrapatna, Parker, Roberts, & Jones, 2020). The data from both scan sessions were aligned to their common midway space using a rigid transformation prior (Maes, Collignon, Vandermeulen, Marchal, & Suetens, 1997).

The spherically averaged signal ${\overset{\circ }{S}}_{\mu }\left(b\right)$ was estimated as the zeroth order spherical harmonic coefficient for each *b*‐value. The spherical harmonic coefficients, up to the sixth order, were estimated using a Maximum Likelihood estimator to account for the Rician data distribution (Sijbers, den Dekker, Scheunders, & Van Dyck, 1998). The spatially varying noise level was estimated prior to the fitting to boost the precision of the estimator (Veraart, Fieremans, & Novikov, 2016).

The diffusion tensor and kurtosis tensor coefficients were estimated by fitting the DKI model to diffusion‐weighted images with *b* ≤ 2.5 ms/μm^2^ using a weighted linear least squares estimator (Veraart, Sijbers, Sunaert, Leemans, & Jeurissen, 2013).

### 2.5 Segmentation

We used tract‐density imaging (Calamante, Tournier, Jackson, & Connelly, 2010) based on whole‐brain probabilistic fiber‐tracking (Tournier, Calamante, Connelly, et al., 2012) of the *b* = 30 ms/μm^2^‐shell to identify all WM voxels using MRtrix 3.0 (Tournier et al., 2019). To minimize partial voluming effects, we retained the top 75% voxels from the tract density map. Moreover, we applied TractSeg for the automated segmentation of individual fiber tracts using the fiber orientation distribution functions as estimated using constrained spherical deconvolution (Wasserthal, Neher, & Maier‐Hein, 2018). The segmented tracts included projection tracts: corticospinal tract (CST), optic radiation (OR); commissural tracts: rostrum, genu, body, and splenium of the corpus callosum; and association tracts: arcuate fasciculus (AF), superior longitudinal fasciculus (SLF), inferior longitudinal fasciculus (ILF), and inferior fronto‐occipital fasciculus (IFO).

### 2.6 Along‐tract analysis

In addition to a voxel‐wise estimation of the effective MR radii, we estimated the effective MR radius along the length of individual tracts, by adopting a novel averaging strategy inspired by methods such as along fiber quantification (AFQ; Yeatman, Dougherty, Myall, Wandell, & Feldman, 2012).

The spherically‐averaged signals were compressed in twenty interspaced segments per white matter tract prior to computing the effective MR radius. For each tract, this compression included the following steps: (a) generate 10,000 tract‐specific streamlines; (2) for each *b*‐value compute the interpolated value of ${\overset{\circ }{S}}_{\mu }\left(b\right)$ in each of the streamline nodes, which must be spaced along each streamline by a distance smaller than the voxel size; (3) compute the center line of the fiber bundle (Klein, Hermann, Konrad, Hahn, & Peitgen, 2007); (4) divide the center line in *N* segments with equal length *L*_*N*_, here *N* = 20. The tangent of the center line in the midpoint of the *i*th segment forms the axis of a cylinder with height *L*_*N*_. The radius of the cylinder equals the maximal distance between an individual streamline and the tangent line within the segment; (5) average the signals of all streamline nodes within the segment‐specific cylinder. To minimize partial voluming with neighboring tissue, the contribution of an individual streamline was weighted by the inverse of its distance to the centerline.

After computing the segment‐averaged ${\overset{\circ }{S}}_{\mu }\left(b\right)$ for each *b*‐value, the effective MR radius can be estimated with a higher precision. Since the spherical mean of the signal is formally a rotationally invariant feature (Mirzaalian et al., 2016), the curvature of the underlying fiber within the segment is assumed not to have a significant impact on the signal averaging and therefore on the estimation.

This presented strategy is also suited to biophysical models that are derived from rotationally‐invariant signal features (Novikov, Fieremans, Jespersen, & Kiselev, 2019; Raven et al., 2020) and that might suffer from poor robustness to noise or partial volume effects.

### 2.7 Statistics

The voxel‐wise test–retest reliability of each diffusion metric *θ* was evaluated per subject and per tract using the test–retest variability and the intraclass correlation coefficient (McGraw & Wong, 1996).

The test–retest variability (TRV) of estimates of parameter *θ* was computed across *N* voxels as:

(3) $$\mathrm{TRV}=\frac{\sqrt{\pi }}{2}\frac{1}{N}\sum_{i=1}^{N}\frac{|\Delta_{\theta }\left(x_{i}\right)|}{\mu_{\theta }\left(x_{i}\right)},$$

with Δ_*θ*_(*x*_*i*_) and *μ*_*θ*_(*x*_*i*_) the difference and the average of the test and retest estimates of parameter *θ* in the *i*th voxel *x*_*i*_, respectively.

The intraclass correlation coefficients (ICC) were calculated for two‐way mixed effects, single measurement, with absolute agreement. ICC estimates were interpreted based on the following guidelines. Values less than 0.5 indicate poor reliability, values between 0.5 and 0.75 indicate moderate reliability, values between 0.75 and 0.9 indicate good reliability, and values greater than 0.90 indicate excellent reliability. ICC values were interpreted considering the 95% confidence interval.

The TRV and ICC were also computed using the nodes derived from our tract‐specific approach instead of the voxels to evaluate the increase in test–retest reliability of along‐tract analysis instead of a voxel‐wise analysis of the dMRI data.

## 3 RESULTS

### 3.1 Whole brain characterization of the effective MR radius

**FIGURE 1:**
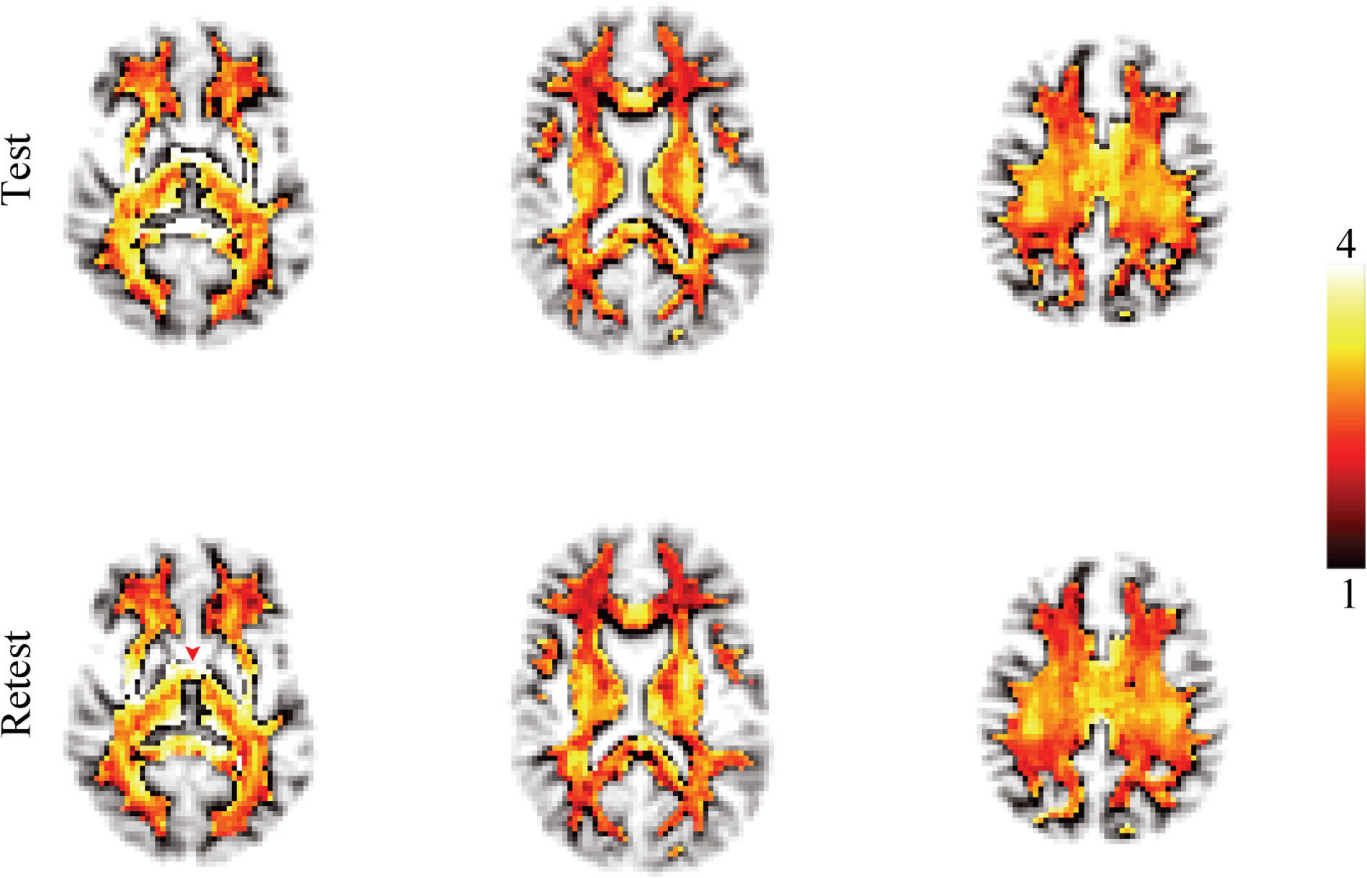
The maps of the effective MR radius (μm) in three slices for the test and retest data. The transparency of the map is set by the tract density to suppress voxels that are not identified as white matter. The red arrow points to the anterior commissure

**FIGURE 2:**
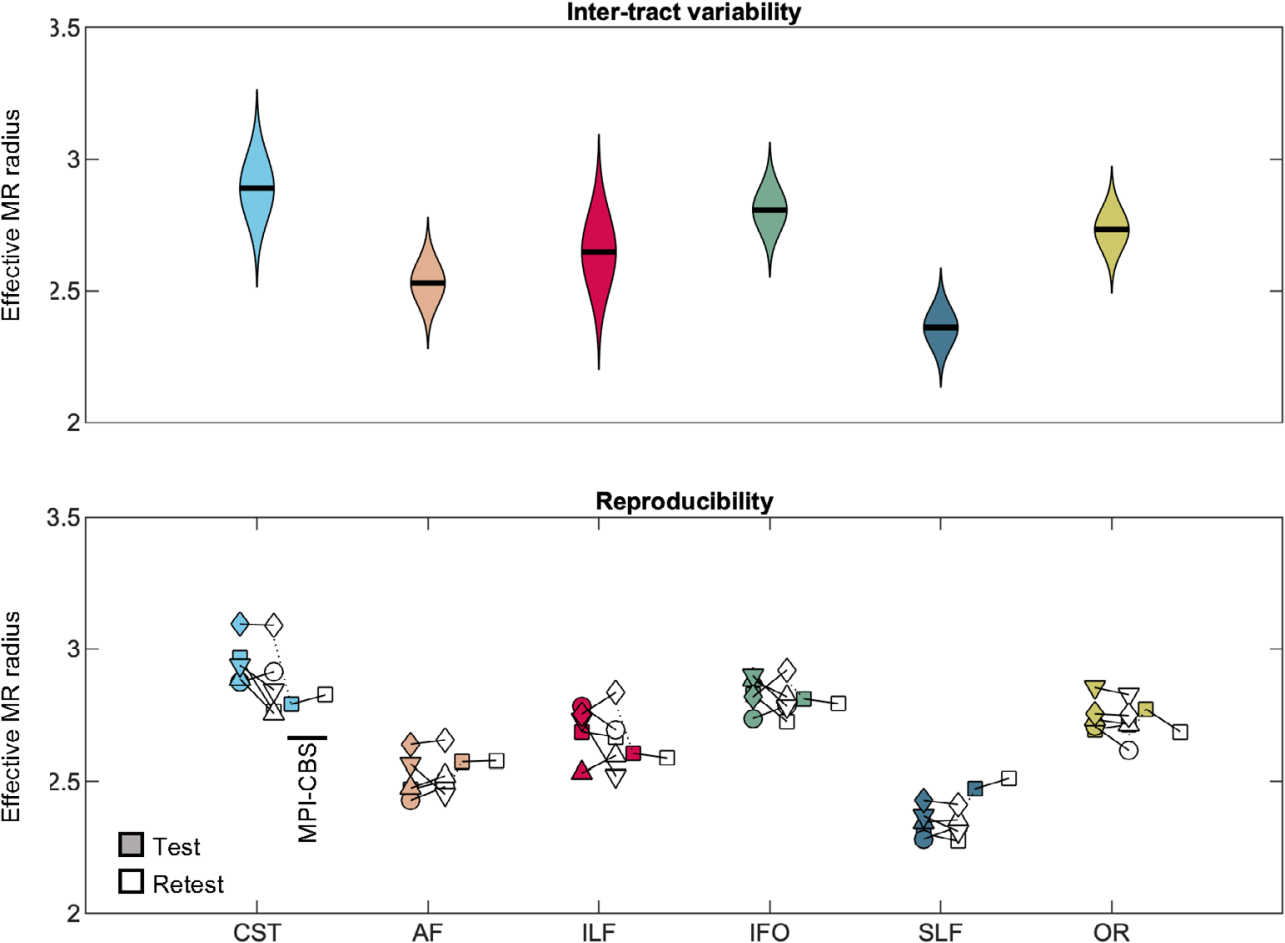
(top) The distribution of MR axon radii (μm). (bottom) The average MR axon radii for each individual subject (markers) is shown per tract for all scan sessions

**FIGURE 3:**
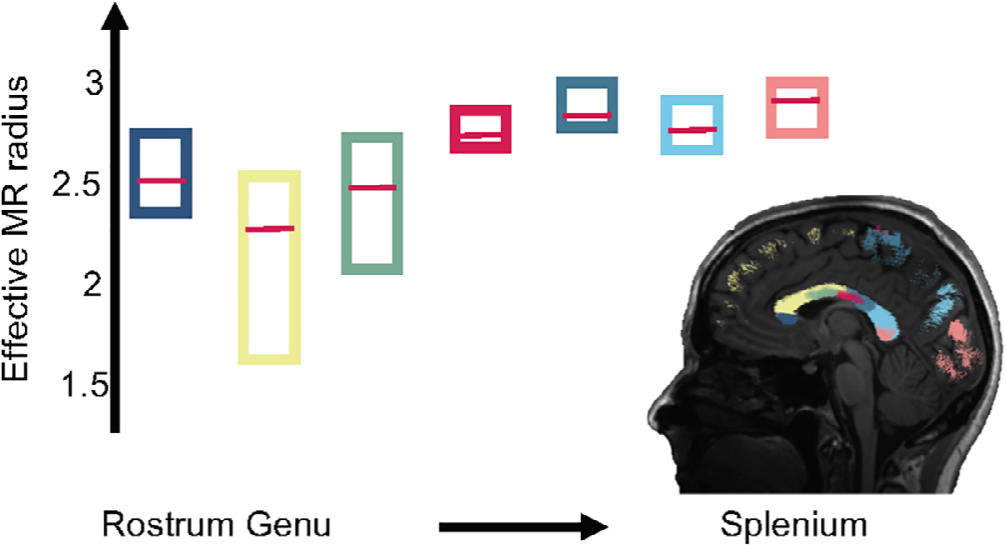
The box plots represent the average effective MR radius (μm) within segments of the human CC, including rostrum (dark blue) and genu to splenium (from left to right), for each of the 5 subjects. The median across the 5 subjects is shown by the red bar, while the boxes cover the 95% confidence intervals. The segmentation of the CC is shown in the inset mid‐sagittal slice

In Figure 1, the voxel‐wise maps of the effective MR radius are shown for 3 slices. The spatial variability of the effective MR radius is apparent, but, overall, the maps are broadly symmetrical. The inter‐tract variability is further highlighted in Figures 2 and 3.

In Figure 2, the distribution of effective MR radius per tract is shown. All voxels of the left and right hemisphere, for all subjects, were considered. In addition, we show the tract‐averaged effective MR radius per subject, and per tract, for the test and retest data to demonstrate that the inter‐tract variability exceeds the inter‐subject variability.

In Figure 3, the trend of the effective MR radius in the mid‐sagittal cross section of the CC is shown. This cross section covers the various segments of the CC, including the rostrum, genu, body and splenium. The box plots show the median and 95% confidence interval of the effective MR radius across the 5 subjects.

**FIGURE 4:**
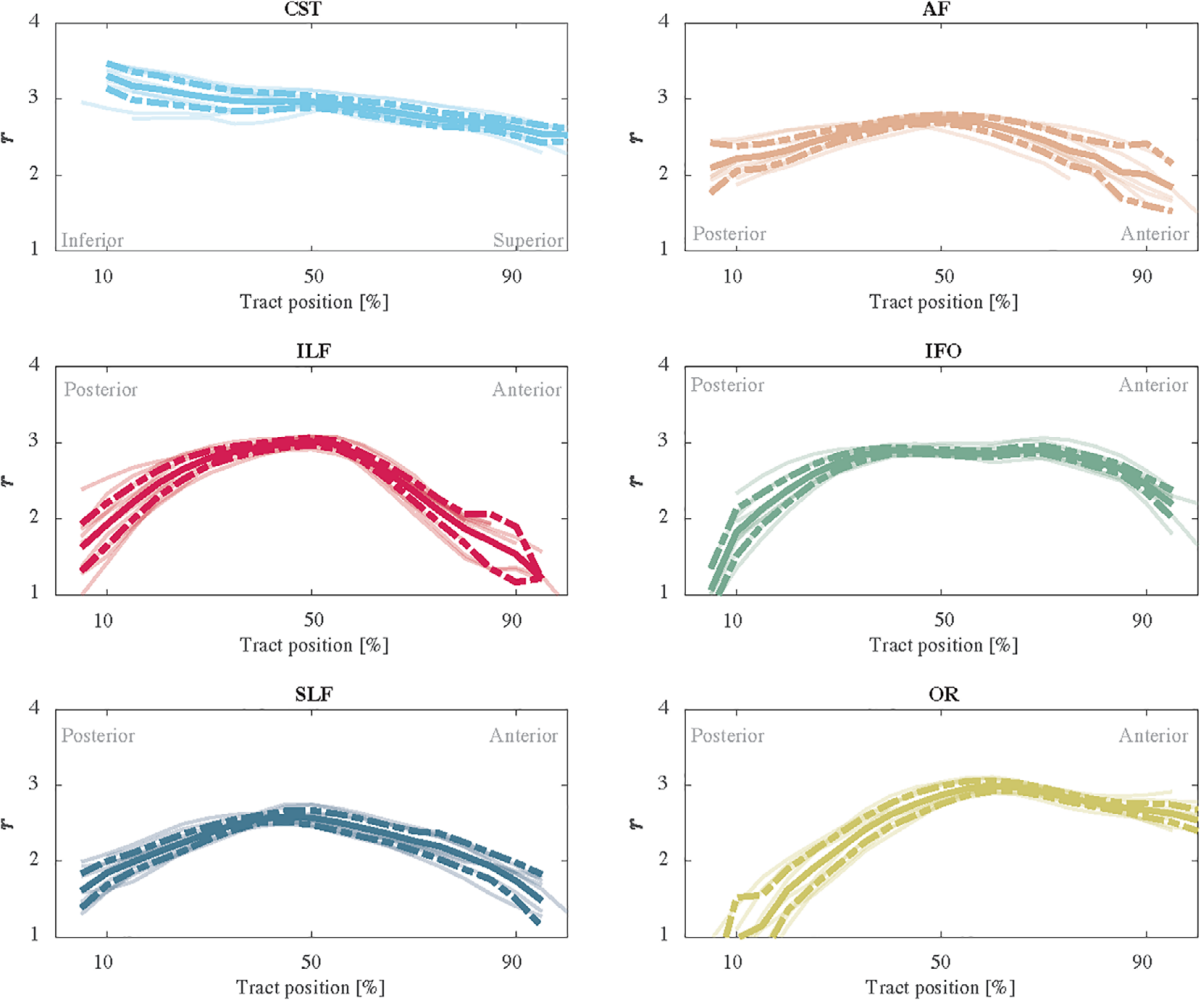
The trend of the effective MR radius *r* (μm) along the tract (posterior to anterior or inferior to superior) for each individual measurements (5 subjects and 2 repetitions) are shown in shaded lines. In addition, we show the average (solid) and 95% confidence intervals (dashed)

In Figure 4, the along‐tract analysis of the effective MR radius is shown. We show the average trend and its confidence interval, computed across all five subjects, including the test and retest data. For completeness, the trends are also shown for each individual subject. The metric changes widely along and across the various tracts.

### 3.2 Correlation matrix

**FIGURE 5:**
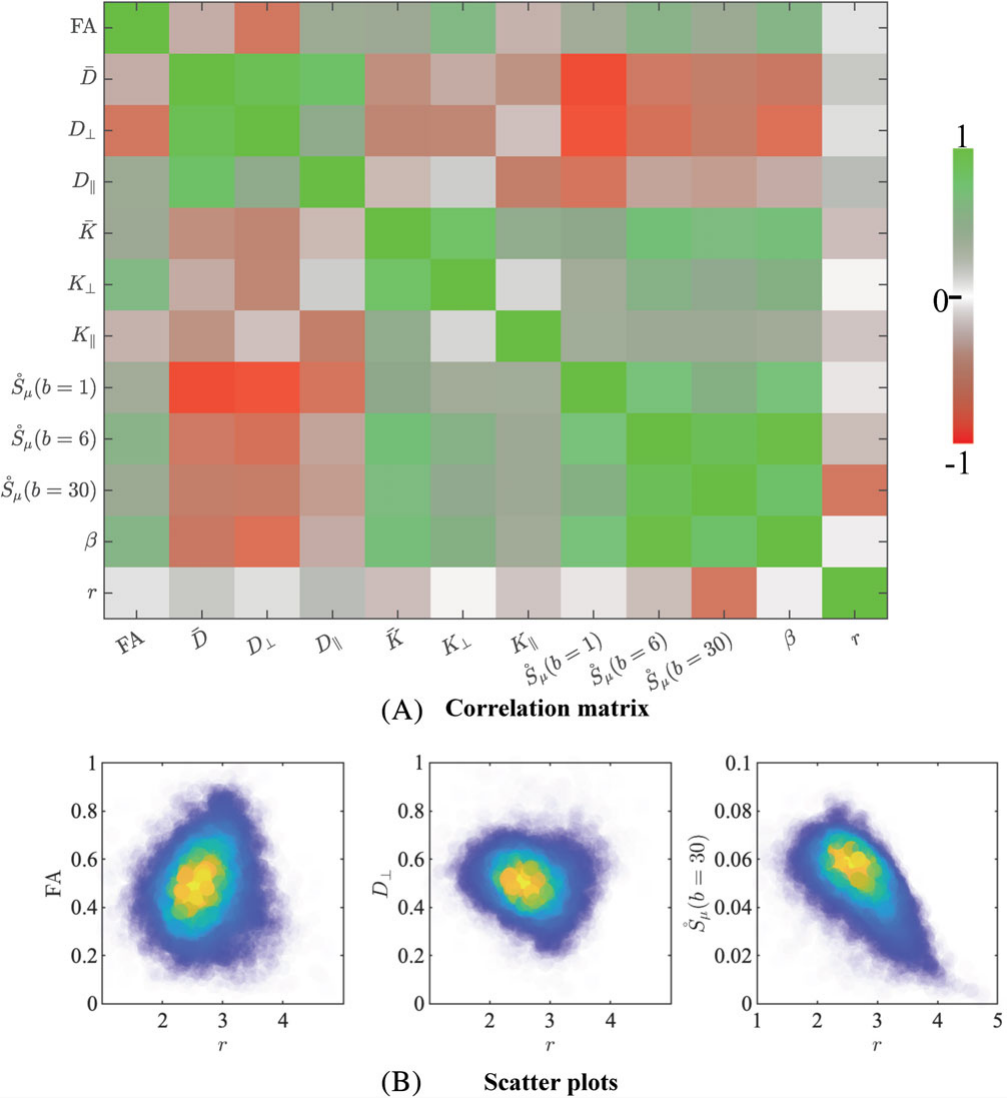
(a) The correlation matrix visualizes the Pearson's correlation coefficient *ρ* that was computed between all pairs of diffusion metrics. Most notably, the effective MR radius *r* shows a minimal correlation with all diffusion metrics. (b) The 2D kernel density plots show the relationship between *r* and FA, *D*_⊥_, and ${\overset{\circ }{S}}_{\mu }\left(b=30\right)$; Blue is low density and yellow is high density

In Figure 5, the correlation matrix shows the Pearson's Correlation Coefficient *ρ* computed between all pairs of diffusion metrics: fractional anisotropy (FA), mean diffusivity ($\bar{D}$), radial diffusivity (*D*_⊥_), axial diffusivity (*D*_∥_), mean kurtosis ($\bar{K}$), radial kurtosis (*K*_⊥_), axial kurtosis (*K*_∥_), ${\overset{\circ }{S}}_{\mu }\left(b=1\right)$, ${\overset{\circ }{S}}_{\mu }\left(b=6\right)$, ${\overset{\circ }{S}}_{\mu }\left(b=30\right)$, *β*, and *r*. The calculation of *ρ* included segmented WM voxels for which all diffusion metric were within biophysically‐plausible bounds. The selected voxels are obtained from both the test and retest data of the five subjects, but are limited to the WM voxels with a tract density exceeding the subject‐specific 75th percentile to minimize partial voluming effects. In total 218,562 voxels were included in the correlation analysis.

In addition, scatter plots show the relation between *r* and FA, *D*_⊥_, and ${\overset{\circ }{S}}_{\mu }\left(b=30\right)$. Overall, the effective MR radius *r* shows no to small correlations with other diffusion metrics. No significant correlations were observed with radial kurtosis *K*_⊥_. A very weak linear relationship ∣*ρ* ∣  < 0.1 was observed for FA, *D*_⊥_, *K*_∥_, ${\overset{\circ }{S}}_{\mu }\left(b=6\right)$, and *β*. The correlation coefficient between *r* and $\bar{D}$, $\bar{K}$, *K*_⊥_, and ${\overset{\circ }{S}}_{\mu }\left(b=6\right)$ was weak with *ρ*= 0.15, −0.18, −0.15, and −0.18, respectively. A strong negative correlation was observed between *r* and ${\overset{\circ }{S}}_{\mu }\left(b=30\right)$ with *ρ* =  − 0.61. This correlation analysis demonstrates that the effective MR radius provides additional information to the various metrics that are more routinely used in dMRI studies.

### 3.3 Test–retest reliability

**FIGURE 6:**
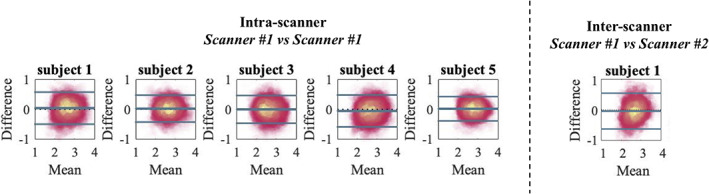
The Bland–Altman shows high reproducibility between scan sessions on the same (left) and different scanner (right) and lack of systematic errors. The solid lines represent mean difference and ±1.96× standard deviations of the difference

In Figure 6, Bland–Altman plots show the agreement between repeated measurements. We show the Bland–Altman plots for the five intra‐scanner test–retest experiments and the single inter‐scanner experiment. For the latter, only the “test” data set from each scanner was considered. The absolute mean difference and its 95% confidence intervals are shown. The percentage differences were 1.51, 1.00, 0.65, −1.67, and 1.17% for the 5 subjects of the intra‐scanner analysis. In comparison, the percentage difference for the inter‐scanner repeatability was −1.19%.

The test–retest reproducibility in the voxel‐wise estimation of the MR axon radius was quantified by the TRV. When including all segmented WM voxels, the TRV varied between 7.83 to 10.48% across the subjects on the first scanner. Hence, the TRV was slightly higher, yet of the same order of magnitude, as conventional DTI metrics (approximately 4% in this study; data not shown). The inter‐scanner reproducibility performance (TRV =10.16%) was similar to the intra‐scanner reproducibility (TRV = 10.48 and 9.04%, for the CUBRIC and MPI‐CBS scanner, respectively).

In Table 1, we list all the TRV per subject and per tract. We observe small fluctuation across tracts, but overall, the test–retest reliability is fairly homogeneous across the brain. A dramatic reduction of TRV, by on average a factor of four is observed, when evaluating the TRV for along‐tract segments instead of voxels. In Table 2, we tabulate the voxelwise ICC for each subject and tract. The test/retest reliability ranged from moderate to good with ICC values ranging between 0.50 and 0.84 with a median value of 0.70. Evaluating the lower bound on the 95% confidence interval did not alter the classification of the reliability. For the approach in which the effective MR radius is computed in a tract‐specific segment instead of voxels, the reliability is good to excellent with a median ICC of 0.84.

**TABLE 1:** The test‐retest variability (TRV; [%]) for the entire WM and individual tracts is tabulated for the voxel‐ and segment‐based analysis for all five subjects

	Voxels							Tract segments						
	WM	CST	AF	ILF	IFO	SLF	OR	WM	CST	AF	ILF	IFO	SLF	OR
1	10.48	7.83	9.89	11.02	10.27	9.68	9.25	2.02	2.53	1.93	2.09	1.34	2.64	1.22
1*	9.04	7.30	8.19	11.78	9.66	6.37	8.88	2.28	1.39	1.67	4.40	1.74	1.62	1.51
2	8.90	5.52	8.11	8.65	8.37	8.21	7.53	2.62	1.90	2.22	3.65	1.37	3.97	1.69
3	9.52	7.03	8.55	12.07	9.39	7.88	9.18	2.56	2.15	3.24	2.75	2.35	1.63	2.92
4	10.05	7.94	9.26	10.97	10.33	8.14	8.79	3.81	2.68	3.71	6.50	3.08	2.28	3.39
5	7.83	4.59	6.71	8.03	7.24	7.16	6.02	1.60	0.92	1.57	1.83	1.49	1.84	1.78

* This subject was scanned on the second scanner (MPI‐CBS) due to the signal averaging of voxels within segment, the TRV of segments is significantly reduced.

**TABLE 2:** The ICC for the entire WM and individual tracts is tabulated for the voxel‐ and segment‐based analysis for the five subjects

	Voxels							Tract segments					
	WM	CST	AF	ILF	IFO	SLF	OR	CST	AF	ILF	IFO	SLF	OR
1	0.71	0.72	0.63	0.73	0.71	0.66	0.70	0.89	0.89	0.96	0.85	0.81	0.96
1*	0.74	0.56	0.70	0.65	0.73	0.77	0.76	0.73	0.94	0.95	0.89	0.84	0.93
2	0.75	0.84	0.70	0.65	0.75	0.77	0.74	0.96	0.86	0.69	0.89	0.63	0.85
3	0.70	0.70	0.65	0.50	0.68	0.69	0.61	0.58	0.74	0.84	0.77	0.92	0.64
4	0.70	0.64	0.69	0.65	0.71	0.74	0.69	0.72	0.79	0.61	0.53	0.85	0.74
5	0.76	0.81	0.72	0.74	0.79	0.73	0.79	0.97	0.64	0.93	0.84	0.90	0.79

* This subject was scanned on the second scanner (MPI‐CBS).

## 4 DISCUSSION

The MR axon radius is a sensitive metric for the *in vivo* and noninvasive detection and quantification of large radii axonal (and possibly glial) projections in human white matter. We demonstrated this by evaluating the along‐ and inter‐tract variability in comparison to repeatability and reproducibility of the metric. In comparison to our previous work (Veraart et al., 2020), we optimized the acquisition protocol in consideration of participant comfort and limitations imposed by *in vivo* research studies by reducing the total acquisition time to 24 min.

The acquisition of two *b*‐shells with strong diffusion‐weighting is required for the accurate and precise estimation of the effective MR axon diameter (see Supplementary material). The precision of the estimator benefits from a wide range of *b*‐values with a minimal value of *b* = 6 ms/μm^2^ to filter out the extra‐cellular signal. We found that *b* = 30 ms/μm^2^ provided a good compromise between *T*_E_ and diffusion time. However, widespread deployment of this technique in human MRI is currently limited by the need for ultra‐strong diffusion‐weighting gradients to achieve these scan settings (Jones et al., 2018; McNab et al., 2013).

**FIGURE 7:**
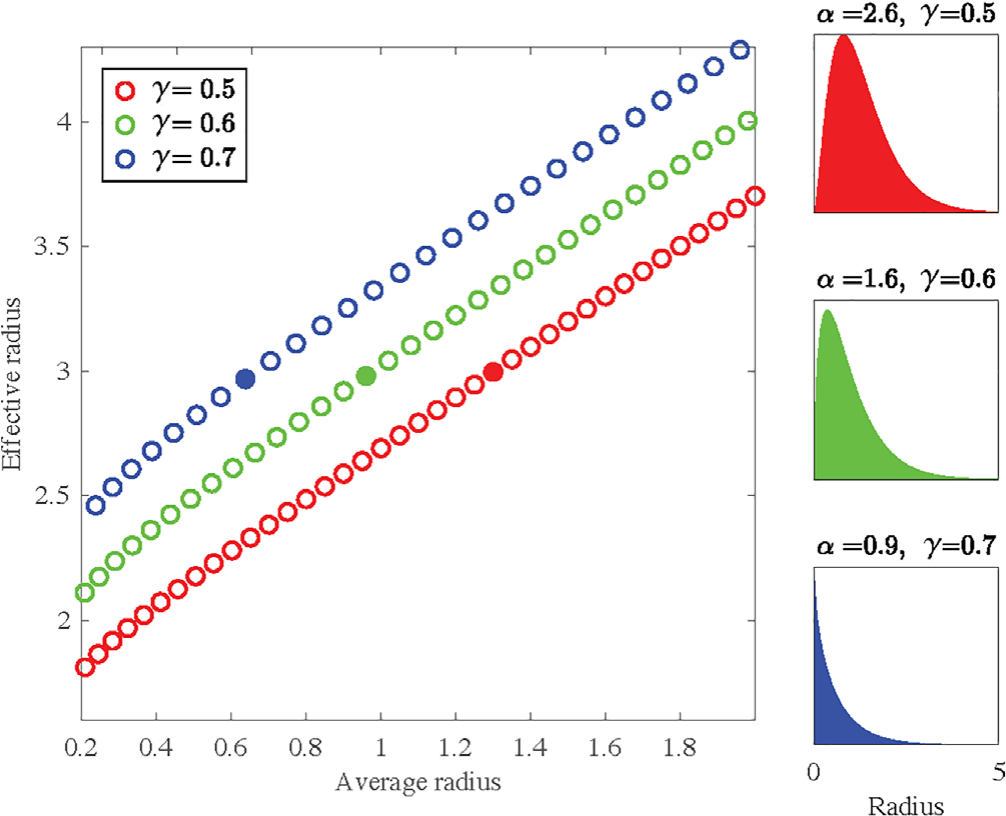
A scatter plot between the average and effective radius of various Gamma distribution with varying shape (*α*) and scale (*β*) parameters are shown. Per color, the scale parameter is fixed. The full distribution is shown for each distribution with an effective radius of 3 (marked by the solid circles). Notably, the corresponding average radii vary widely

The effective MR axon radius estimation is intrinsically sensitized towards large axons; therefore, it is not representative of the full distribution of axon radii present in a voxel (Alexander et al., 2010; Burcaw et al., 2015; Veraart et al., 2020). If it were, it would be much more straightforward to compare and validate with microscopy data. Sepehrband, Alexander, Clark, et al. (2016) studied the accuracy of various parametric distributions to describe the axon distribution in the mouse corpus callosum. All well‐fitting distributions were described by at least two parameters (Sepehrband, Alexander, Clark, et al., 2016), so trying to reconstruct the parametric distribution from the effective MR radius alone is ill‐posed. This problem is highlighted in Figure 7 where we show the relation between the average and effective radius of Gamma distributions with varying shape *α* and scale *γ* parameters. In order to obtain a unique mapping from the effective to the much lower average radii, at least one parameter, *α* or *γ*, must be known or chosen *a priori*. Unfortunately, distributions with different *α* and *γ* result in the same effective radius, with a widely varying average radius, while the corresponding shape of the distribution are all plausible candidates in describing realistic axon distributions. When fitting a Gamma distribution to the distributions shown in Wegiel et al. (2018), we conclude that the shape of the axon radius distribution, both in terms of *α* or *γ*, is significantly different between typically‐developing children and children with ASD. This prevents us from fixing one of the distribution parameters and, as such, from mapping the effective to the average radius accurately.

On the bright side, the effective MR radius might be a very sensitive metric to distinguish between cohorts. Based on data reported in the postmortem study of Wegiel et al. (2018), we estimate that the percentage difference in effective MR radius is about 18% in the splenium of the corpus callosum when comparing typically developing children and children with ASD. With a voxelwise TRV of about 10%, one would only need a total sample size of N=14, with equal representation of both cohorts, to detect the difference in the effective MR radius with a statistical power of 0.96 (Faul, Erdfelder, Buchner, & Lang, 2009; Lakens, 2013). This preliminary power analysis highlights the feasibility of MR axon radius mapping in future research studies. The good inter‐site agreement also bodes well for the future inclusion of effective MR diameter mapping in studies of rare or difficult‐to‐recruit disorders or diseases, where multi‐site studies are needed to achieve the necessary statistical power to make a robust inference about a given pathology (cf. Jahanshad et al., 2013).

The test–retest reliability was good to excellent when adopting the along‐tract analysis (Yeatman et al., 2012). This strategy is perfectly suited to the technique because the data are rotationally invariant along tract segments. As segments are averaged prior to model fitting, there is no loss of accuracy due to the underlying curvature of the tract (Mirzaalian et al., 2016). With a TRV of only a few percent, even in an inter‐scanner evaluation, the sensitivity of the MR axon radius mapping was able to detect subtle changes across the brain, both inter‐ and along‐tract within a single subject. Therefore, the technique is sensitive down to the individual segments. This is a potential limitation for very local effects; however, Yeatman, Wandell, and Mezer (2014) found sensitivity to lesions in patients with multiple sclerosis using along tract segments.

Moreover, “spherically‐averaging” modeling approaches, such as the axon diameter mapping presented here and others (Kaden, Kruggel, & Alexander, 2016; Novikov, Veraart, Jelescu, & Fieremans, 2018; Reisert, Kellner, Dhital, Hennig, & Kiselev, 2017) entail an implicit assumption that dMRI data are perfectly shelled; that is, different gradient directions exist for a finite set of *b*‐values. This assumption is usually unmet due to gradient nonlinearities and poses an unnecessary constraint on experimental design (Bammer et al., 2003). Although we corrected for the gradient nonlinearities by a spatially dependent scaling of the *b*‐values, we could not account for the potential directional variability of the gradient nonlinearities due to the need for shelled data (see Afzali, Knutsson, Özarslan, and Jones (2020)). Alternatives to the spherical mean may well prove useful in the future, but is beyond the scope of the current work.

The consistent tract variability of the estimated effective MR radius within and between subjects is in agreement with histological data across various species. The observed “low‐high‐low” trend in axon radii across from the genu to splenium of the CC is in fair agreement with the variations in axon radius distributions previously reported in rat (Barazany, Basser, & Assaf, 2009; Sargon et al., 2003; Veraart et al., 2020), rhesus monkey (LaMantia & Rakic, 1990), and human (e.g., Aboitiz et al., 1992). The slightly larger radii in the rostrum of the corpus callosum are in qualitative agreement with Sargon et al. (2003). Overall, this result demonstrates that the effective MR radius detects differences in axon radius distribution across the CC. Furthermore, the large radii of the CST, in comparison to other tracts, have been reported extensively in the literature (Tomasi et al., 2012). However, the comparison of MR to histology is challenged by the need for the full axon radius distribution, which is usually not reported.

The consistent inter‐tract variability was also observed in the recent work of Huang et al. (2020). However, despite some common findings, we did not observe an anterior–posterior gradient in effective MR radii. The difference in results might be rooted in modeling choices. In our approach, we aim to minimize modeling assumptions by using the acquisition itself, that is, high *b*‐value, to filter out both extra‐cellular signal and orientational dispersion prior to modeling the intra‐axonal signal.

Even after eliminating orientational dispersion, it is not understood why we observe such strong along‐tract variability of the MR axon radii mapping in certain tracts (e.g., CST). Various additional confounding factors have been discussed, for example, diameter variations due to curvature or undulations of the axons (Andersson et al., 2020; Brabec, Lasič, & Nilsson, 2020; Lee, Jespersen, et al., 2020; Lee, Paioannou, Kim, Novikov and Fieremans 2020; Nilsson, Lätt, Ståhlberg, van Westen, & Hagslätt, 2012). However, in agreement with our previous hypothesis, this observation might also be explained by the lack of specificity of the high *b* signal to axons only. The density and variability of cells in the human brain is considerable. It has been observed that the numerous and dispersed glial processes, from astrocytes in particular, are abundant in the white matter (Luse, 1956; Oberheim et al., 2009; Perge et al., 2009) and have radii that exceed even the largest axons (Oberheim et al., 2009). Even a relatively small fraction of these large glial processes might bias the MR axon diameter mapping due to the nature of how signals are encoded.

In comparison with other regions identified from the whole brain analysis, the MR axon radius is systematically very high in the most anterior interhemispheric connections of the white matter, see Figure 1. (LaMantia & Rakic, 1990) performed an in‐depth cytological characterization in rhesus macaque of these tissues, identifying a functionally distinct sub‐region of the anterior commissure ‐ the basal telencephalic commissure. The axon radii distribution for these interhemispheric projections is comprised primarily of small axons; however, the basal telencephaplic commissure is encapsulated by glial processes remaining from neural migration in early fetal development (Lent, Uziel, Baudrimont, & Fallet, 2005). These glial cells have been identified as GFAP‐positive fibrous astrocytes, and might contribute to the high axon radii estimates in that region.

MRI spectroscopy may offer some additional insight towards the hypothesis that MR axon diameter mapping, and the interpretation of biophysical modeling using MRI in general, might be influenced by glial cells. In Palombo, Ligneul, and Valette (2017), the effective MR radius of glial processes was potentially larger than neuronal processes as shown with diffusion‐weighted signal decay at high *b*‐values for various metabolites. In along‐tract analysis of metabolites, the ratio of Choline (Cho) to N‐acetyl aspartate (NAA) had similar trends to MR axon radius in the CST (Govind et al., 2012). NAA is highly concentrated in neurons, whereas Cho have been shown to originate predominantly from glial cells. Further investigation is needed to disentangle these effects, which may be aided by tissue regions with high levels of naturally present glial cells, such as the basal telencephalic commissure.

## 5 CONCLUSION

We demonstrated a good to excellent reliability in the quantification of micron‐sized effective MR radii using human dMRI if ultra‐strong diffusion‐weighted gradients are employed. As a result, we were able to observe the subtle inter‐ and along‐tract variability that has previously been reported in histological studies. However, our results foster the hypothesis that dMRI signals at high *b*‐values might not be exclusively sensitive to neuronal processes or axons and that the contribution of glial processes to the dMRI signal needs to be better understood to allow for an unambiguous interpretation of morphological parameters such as the effective MR radius.

## CONFLICT OF INTEREST

The Max Planck Institute for Human Cognitive and Brain Sciences has an institutional research agreement with Siemens Healthcare. NW was a speaker at an event organized by Siemens Healthcare and was reimbursed for the travel expenses. NW holds a patent on acquisition of MRI data during spoiler gradients (US 10,401,453 B2).

## Supporting information


**Figure S1** (a) The distribution *P* of sampled *b*‐values for a protocol with uniform sampling in *G* (blue) and a CRLB optimized protocol (green). For the optimization, *δ*, Δ, and *r* were fixed to 15 ms, 30 ms, and 2 μm, respectively. Little variability was observed when varying those settings. (b) The optimization landscape of the CRLB as a function of *δ* and Δ. The cross hair shows the combination of *δ* and Δ that were selected in our study. The 1D optimization landscapes at that intersection are also shown (right). The dashed white line represents all combinations of *δ* and Δ with an equal precision of the estimator of the effective MR radius—when ignoring *T*_*E*_ dependencies.

**Appendix** **S1**: Supplementary material

## Data Availability

The data from this study are available from the corresponding author upon reasonable request.
