# Supplementary figures and images for: The variability of MR axon radii estimates in the human white matter
Source: Hum Brain Mapp. 2021 Feb 12;42(7):2201–13. doi: 10.1002/hbm.25359 (PMC8046139; doi:10.1002/hbm.25359)

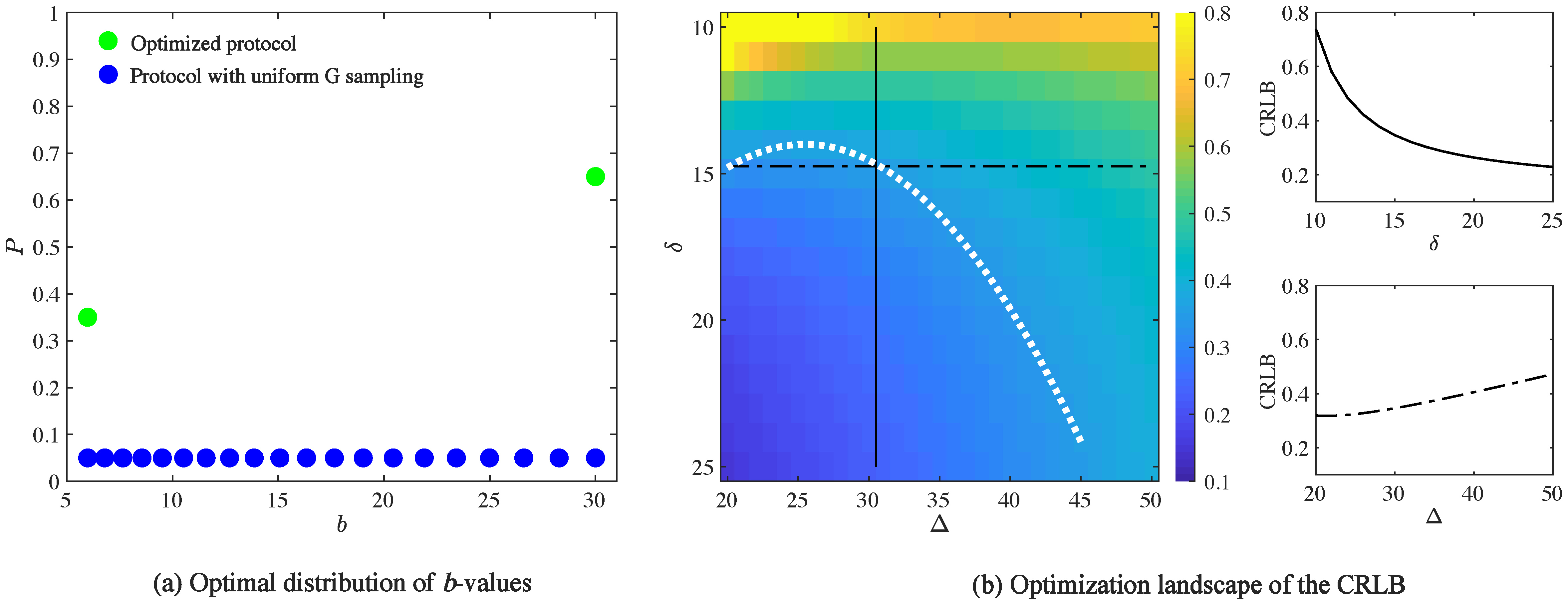

Supplement: Supplementary file 1 — Figure S1 (a) The distribution P of sampled b‐values for a protocol with uniform sampling in G (blue) and a CRLB optimized protocol (green). For the optimization, δ, Δ, and r were fixed to 15 ms, 30 ms, and 2 μm, respectively. Little variability was observed when varying those settings. (b) The optimization landscape of the CRLB as a function of δ and Δ. The cross hair shows the combination of δ and Δ that were selected in our study. The 1D optimization landscapes at that intersection are also shown (right). The dashed white line represents all combinations of δ and Δ with an equal precision of the estimator of the effective MR radius—when ignoring TE dependencies. [file HBM-42-2201-s002.tiff]
