# Supplementary material for: The variability of MR axon radii estimates in the human white matter
Source: Hum Brain Mapp. 2021 Feb 12;42(7):2201–13. doi: 10.1002/hbm.25359 (PMC8046139; doi:10.1002/hbm.25359)
Supplement: Supplementary file 2 — Appendix S1: Supplementary material [file HBM-42-2201-s001.docx]

## Supplementary material

### *Considerations for experimental design*

In stark contrast to our previous work, our current protocol for axon diameter mapping has only two distinct $b$-values – the bare minimum to solve Eq. 1. We will explain here that such a *simple* protocol results in a significantly elevated attainable precision in the estimation of the effective MR radius compared to protocols with more densely or uniformly sampled $b$-values.

The following constraints are imposed: (a) The diffusion gradient duration $\delta$ and separation $\Delta$ must be constant across the $b$-values to exclude the time-dependency of $D_{a}$ as a potential confounding factor (Lee et al., 2020b); (b) All $b$-values must exceed $6 \text{ms/μm}^{2}$ to suppress the extra-cellular signal (Veraart et al., 2019).

The following questions remain to be answered: (1) What is the optimal distribution of gradient strengths $G$ (or b-values)?; and (2) What are the optimal gradient timings $\delta$ and $\Delta$ for axon diameter mapping? We here adopt a two-step strategy using a Cramér-Rao Lower bound (CRLB; Aitken and Silverstone, 1942) on the variance of the estimator of the effective MR radius to gain insight into the *optimal* protocol. Although the simultaneous optimization of $G$, $\delta$, and $\Delta$ might be more elegant, it has less practical value because the scanner and scan sequence imposes various practical limitations that are often challenging to model analytically.

First, the CRLB analysis demonstrated that the acquisition of only two distinct $b$-shells is favorable in comparison to the previously used protocol, e.g. uniform sampling in $G$ – see Figure S1(a). The optimal $b$-values are the bounds of the available $b$-range, with a lower bound of $b=6 \text{ms/μm}^{2}$ and a maximal $b$-value that depends on the maximally available gradient strength. However, the higher $b$-shell must be sampled more often – more gradient directions or more repetitions need to be acquired. Our CRLB analysis showed that the optimal ratio between the number of diffusion-weighted images at each $b$-value varied between 1.88 and 2.29 for radii ranging between 1 and 3 $\text{μm}$. In comparison to a uniform sampling in terms of $G$, we observed that the maximally attainable precision increased from 60 to $63\%$ when adopting the two-shell protocol for axon diameter mapping.

Second, the CRLB analysis also demonstrated that increasing $\delta$ and/or decreasing $\Delta$ improves the precision of the parameter estimator – see Figure S1(b). Although increasing $\delta$ is the most efficient way to improve the estimator’s precision, it comes with a high penalty in echo time $T_{\text{E}}$ and $\Delta$. The translation of the theoretical insights into actual scan settings required a heuristic approach in our study to account for the practical limitations of the scanner and the sequence. We recommend the following guidelines. It is important that the gradient duration $\delta$, separation $\Delta$, and magnitude $G$ are tuned to a minimal sensitivity to the axon diameter at $b=6 \text{ms/μm}^{2}$ and a maximal sensitivity at the second $b>6 \text{ms/μm}^{2}$-shell. To realise these conditions in practice, for a given $T_{\text{E}}$, we first maximized $\Delta$ (and $\delta$) to achieve $b=6 \text{ms/μm}^{2}$ with minimal $G$. Next, we maximized $G$ while keeping the $\Delta$ and $\delta$ constant to achieve our highest attainable $b$-value.

A further improvement to this optimization strategy can be made by including the $T_{\text{E}}$ as a function of $\delta$ and $\Delta$ to penalize excessive $T_{2}$-weighed signal loss.

Figure S1: (a) The distribution $P$ of sampled $b$-values for a protocol with uniform sampling in $G$ (blue) and a CRLB optimized protocol (green). For the optimization, $\delta$, $\Delta$, and $r$ were fixed to 15$\text{ms}$, 30$\text{ms}$, and 2$\text{μm}$ respectively. Little variability was observed when varying those settings. (b) The optimization landscape of the CRLB as a function of $\delta$ and $\Delta$. The cross hair shows the combination of $\delta$ and $\Delta$ that were selected in our study. The 1D optimization landscapes at that intersection are also shown (right). The dashed white line represents all combinations of $\delta$ and $\Delta$ with an equal precision of the estimator of the effective MR radius – when ignoring $T_{E}$ dependencies.
